# Complicated malaria symptoms associated with *Plasmodium vivax* among patients visiting health facilities in Mendi town, Northwest Ethiopia

**DOI:** 10.1186/s12879-016-1780-z

**Published:** 2016-08-22

**Authors:** Yohannes Demissie, Tsige Ketema

**Affiliations:** Department of Biology, College of Natural Sciences, Jimma University, Jimma, Ethiopia

**Keywords:** Hemoglobin, Hypoglycemia, *P. vivax*, Respiratory distress, Severe malaria

## Abstract

**Background:**

Malaria is still a major health problem in some parts of the world. *Plasmodium falciparum* is the common pathogenic parasite and is responsible for majority of malaria associated deaths. Recently the other benign parasite, *P. vivax,* is reported to cause life threatening severe malaria complications. Thus, this study was aimed to assess incidence of severe malaria symptoms caused by *P. vivax* parasite in some malaria endemic areas of Ethiopia.

**Materials and methods:**

Presumptive malaria patients (all age groups) seeking medication at the selected health facilities in Mendi town, Northwest Ethiopia, were recruited for the study. Socio-demographic, clinical and parasitological characteristics were assessed following standard procedures. Data was analyzed using descriptive statistics, chi-square test and relative risk.

**Results:**

Of the 384 patients enrolled in the study for *P. vivax* mono-infection, 55 (14.3 %) of them were fulfilled at least one of the WHO criteria for severe malaria indicators. Some of these clinical manifestations were: prostration 14 (25.45 %), persistent vomiting 9 (16.36 %), respiratory distress 6 (10.9 %), hypoglycemia 5 (9.1 %), hyperpyrexia 8 (14.5 %), and severe anemia 13 (23.63 %). Differences in parasite load did not affect the frequency of some severe malaria symptoms. However, severe anemia, prostration, and persistent vomiting were significantly affected (*P* < 0.05) by relatively higher load of parasitemia, (OR = 3.8, 95 % CI, 1.1–13.7; OR = 4.4, 95 % CI, 1.4–13.9; and OR = 7, 95 % CI, 1.8–27.4) respectively.

**Conclusion:**

*P.vivax* associated severe malaria symptoms observed in this study is supportive evidence for the notion that *P.vivax* is no longer benign parasite but rather virulent. Thus, to meet international and regional targets of malaria eradication, a holistic prevention and control approaches should be designed.

## Background

Malaria is one of the most important health problems in developing countries. It is estimated that about half of the world’s population are at risk of malaria [1, 2]. *P. falciparum* and *P. vivax* have worldwide distribution, with *P. falciparum* being the more pathogenic. Few years back, it was indicated that about 1–3 million mortality per year, mainly in children and pregnant women, are due to severe malaria caused by *P. falciparum* [3]. However, according to the latest report released by WHO [4] there were an estimated 584,000 deaths globally. The same report showing reduction of malaria mortality rates among children in Africa by an estimation of 47 % globally since 2000 and by 54 % in the WHO African Region [4].

In Ethiopia malaria is unstable and seasonal. This is because of the country’s heterogeneous topography and climatic factors [5, 6]. Areas at altitudes between 1600 and 2000 m above sea level (masl) are epidemic-prone hypo-endemic zones of malaria [7]. However, malaria epidemics are expanding to areas as high as 2500 masl [8]. On the other hand, although *P. vivax* is a rare parasite in most parts of Africa, it is an important parasite in Ethiopia. In some areas of the country the prevalence rate even exceeds 70 % of total malaria infections. This was previously reported due to the high Duffy positivity trait of most population of Ethiopia, but recently contradictory reports are coming [9–11]. In addition, chloroquine resistance pattern of *P. vivax* parasite is increasing in the country [12]. Recently severe life threatening malaria symptoms*,* frequently associated with *P. falciparum,* has been reported from Asia, South America and Africa for *P. vivax* [13–18]. Thus, the current study was aimed to assess incidence of severe malaria symptoms due to *P. vivax* infection in one of malaria endemic areas of Ethiopia.

## Methods

### Description of the study area

**Fig. 1:**
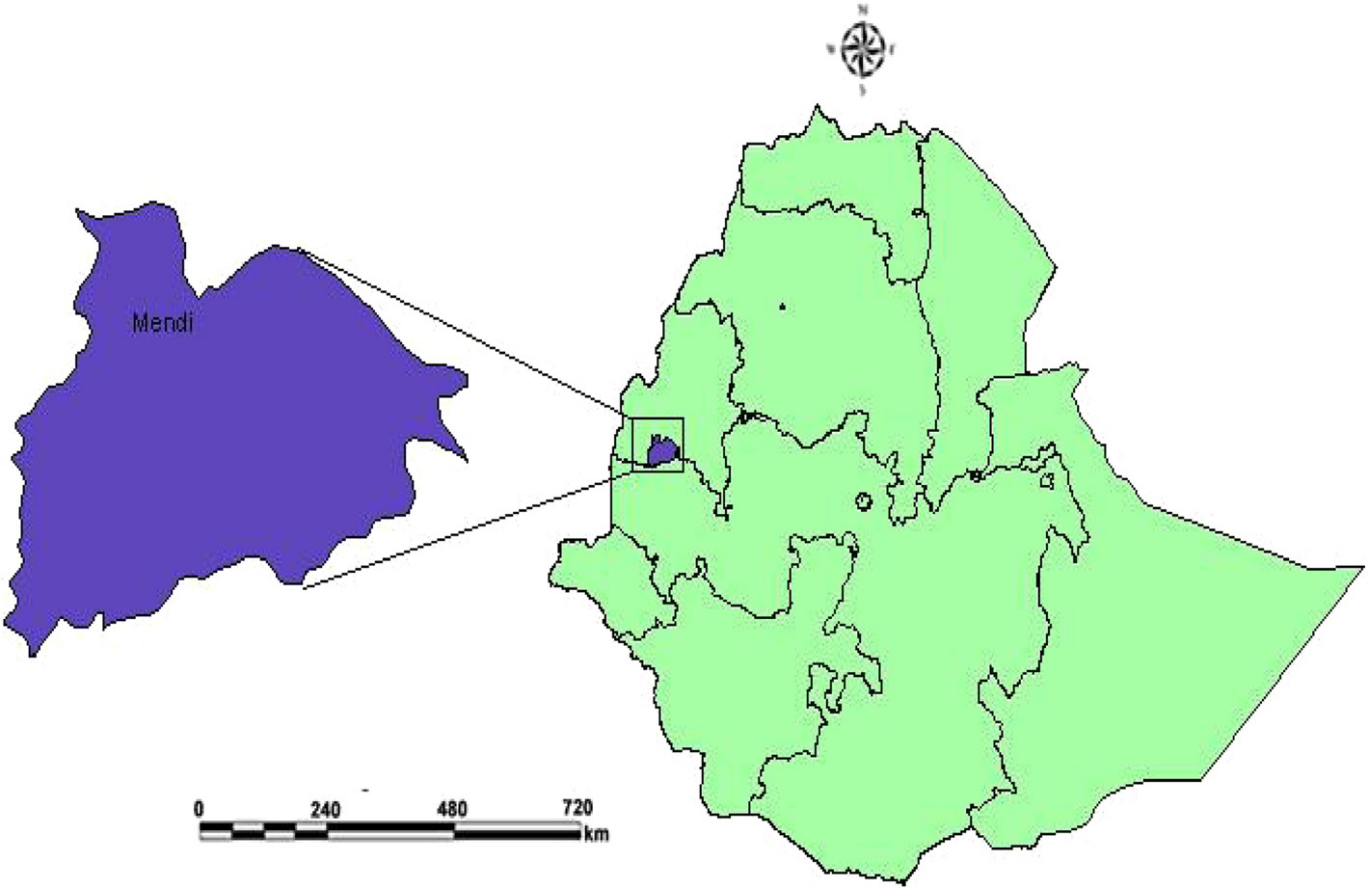
Map of the study site

A cross sectional study was conducted in Mendi Town, located 567 km Northwest of Addis Ababa, the capital city of Ethiopia (Fig. 1). Geographically the study site is located at latitude and longitude of 9°48′N 35°6′E and the mean altitude of 1538 masl. Annual rainfall ranged from 900 to 1500 mm and main rainy season is from May to October. The mean annual maximum temperature of the area is about 32 °C (25–32). The two principal parasites of malaria infection in the study area are *P. falciparum* and *P. vivax*. The main vector in the study area is *A. arabiensis*. Based on the 2007 Census conducted by the Central Statistical Agency (CSA) of Ethiopia, this town has a total population of 28,485 of whom 14,385 are males and 14,100 females [19].

### Study population

The study participants were all presumptive malaria patients seeking medication at the health center and clinic in Mendi town, during the study period from September, 2014 to June, 2015 and those who had symptoms of malaria infection. The inclusion criteria used were any patient positive for *P. vivax* mono-infection (microscopy confirmed), without any chronic illness, without prior medication for the current illness, having clinical symptoms such as fever, chills, malaise, headache, vomiting, history of fever for about 48 h before admission, and volunteer to participate in the study. Accordingly, a total of 384 patients who fulfilled the inclusion criteria were included in the study.

### Data collection

Clinical and demographic data were collected using pre-designed case record form by trained health professionals working in the two health facilities. Clinical symptoms such as fever, headache, diarrhea, hyperpyrexia, hemoglobunria, persistent vomiting, impaired consciousness, convulsion, respiratory distress, persistent vomiting, hypotension, anorexia, nausea, and rigor were assessed following criteria set on guideline for malaria diagnosis and treatment [20]. Body temperature of each patient was measured using digital thermometer.

Patients with at least one symptom of severe malaria complications set by World Health Organization (WHO) were classified as severe *P. vivax* cases [20]. Preliminary screening of the blood smears for *P.vivax* was done by two experienced laboratory technician working in the two health facilities. To ensure data quality, the positive slides were further confirmed by certified senior laboratory technician at Jimma University. Fortunately there were no difference in their reading and the original record was used for analysis. Detailed description of the blood collection and smear preparation is as follows: a drop of blood sample was collected on clean glass slide from lancet pricked finger to prepare thin and thick blood smears in duplicate per patient for microscopic examination. Thick and thin blood smears were stained with 3 % Giemsa (pH = 7.2, for 45 min), while thin smears were fixed in methanol before Giemsa staining. Malaria parasite was identified by observation of the smears and the morphological appearance of the parasite in the infected red blood cells (RBCs) using ×100 microscope objective. Parasite load was calculated after counting asexual parasites per 200 white blood cells (WBC), assuming mean WBC count of 8000 count/μL using the following formula:

$$ \mathrm{Parasite}\kern0.5em \mathrm{load}/\upmu \mathrm{L}=\frac{\mathrm{Number}\kern0.5em \mathrm{of}\kern0.5em \mathrm{observed}\kern0.5em \mathrm{asexual}\kern0.5em \mathrm{parasites}\times 8000\kern0.5em \mathrm{W}\mathrm{B}\mathrm{C}\kern0.5em \mathrm{count}/\upmu \mathrm{L}}{200\kern0.5em \mathrm{WB}\mathrm{C}\mathrm{s}} $$

The degree of parasitaemia was graded as mild, moderate, and severe, when a count (x) is between 1 and 999 parasite/μL, 1000–9999/μL, >10,000/μL, respectively [21].

From the pricked finger, some drops of blood samples were taken for quantification of levels of blood glucose (Glu) (*Senso Card* Hungary) and hemoglobin (Hb) (Hemocue™, haemoglobino meter, Angelholm, Sweden, Hb 301). The patient was considered anemic when Hb level is <11 g/dL (children) and <13 g/dL (adult male) and <12 g/dL dult female). Further, level of anemia was classified as severe, moderate and mild, when Hb level is <5 g/dL, between 5 and 8 g/dL, and between 8 and 11 g/dL, respectively [20]. Lower blood glucose (hypoglycemia) was considered when blood glucose concentration was <40 mg/dL. Hyperpyrexia was considered when body temp is ≥40 °C. All participants were treated with Chloroquine sulfate as soon as the blood sample was drawn and found positive for *P.vivax* following National Guideline for Malaria Diagnosis and Treatment [22].

### Data analysis

Data was analyzed using SPSS statistical software (Version 20.0. Armonk, NY: IBM Corp). Descriptive statistic was used for analysis of some clinical, demographic and parasitological data. Associations between variables were computed using Pearson correlation. Responses were compared using chi-square test, and relative risk was used to assess strength of association between variables in groups. In all analysis, significance level was considered at 95 % confidence interval (CI).

## Results

### Prevalence of malaria in the study area

**Fig. 2:**
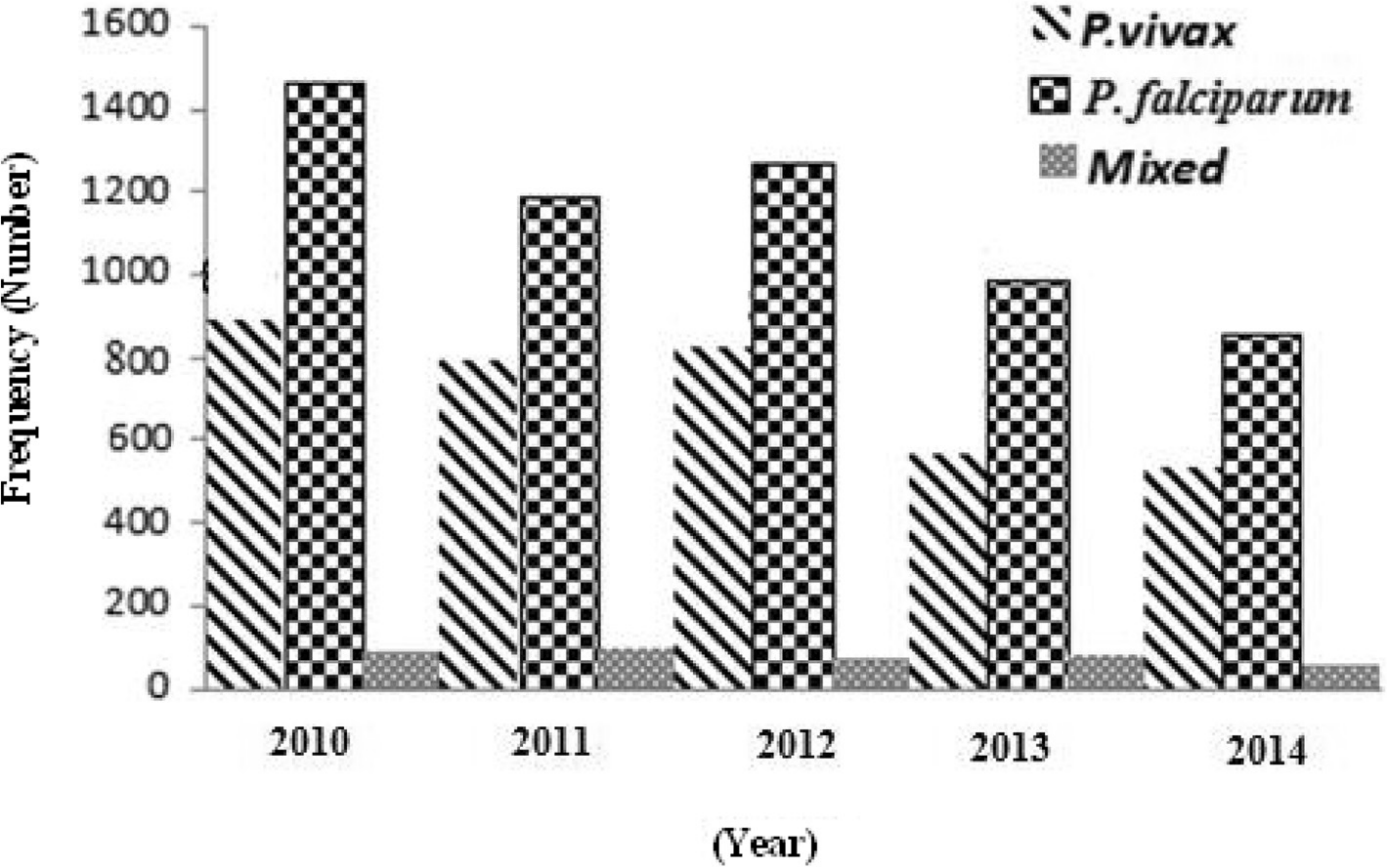
Malaria cases among patients in Mendi town, Oromia, Northwest Ethiopia, 2010–2014

The trend of malaria cases showed an irregular declining pattern. However, the 5 years prevalence report (2010–2014) of the two health facilities showed that, the number of malaria infected patients was still higher (Fig. 2).

**Fig. 3:**
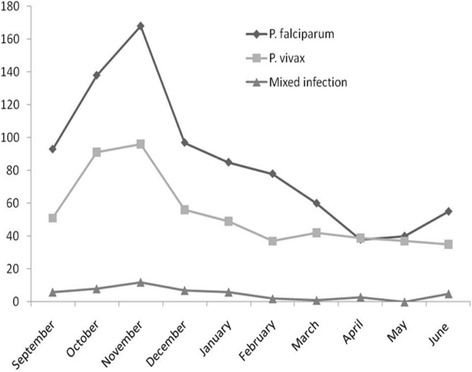
Confirmed malaria cases during the study period at health facilities in Mendi town, Northwest Ethiopia, 2014/15

Of the total 14, 844 individuals (8751 females and 6093 males) seeking medication at the two health facilities, blood samples were collected from 4813 suspected malaria cases. Accordingly, 1434 (818 males and 616 females) were found positive for malaria infection. Among the positive cases, A total of 533 (37.2 %) and 851 (59.3 %) were infected with *P. vivax* and *P. falciparum*, respectively, while, 50 (3.5 %) were due to mixed infection (*P. vivax* and *P. falciparum*). The highest peak infection for *P. falciparum* and *P. vivax* was observed in November followed by October (Fig. 3).

### Socio-demographic and clinical characteristics of the study participants

In the current study, a total of 384 patients that fulfilled the inclusion criteria were clinically diagnosed and enrolled in the study. Prevalence of malaria among male and females patients were 52.6 % (*n* = 202) and 47.4 % (*n* = 182), respectively. Among different age groups, the distribution of malaria infection with respect to sex was almost similar, except in those aged less than 5 years, where the infection was higher among male population of the same age. In terms of sex, male malaria patients (*n* = 202, 52.6 %) were significantly higher than that of females (*χ*^2^ = 64.2, *P* < 0.05).

Median age of the study participants was 14 years (1 month to 60 years). Mean body temperature and blood glucose levels of the study participants were 37.89 °C (35.5 to 40.8) and 104.4 mg/dL (36.8 to 180), respectively. Also, the level of Hb and geometric mean parasite counts were 12.4 g/dL (4.3–17.8) and 3745parasite/μL (280–31,600), respectively. The observed anemic cases were 115 (29.9 %) (Table 1).

**Table 1:** Clinical and demographic characteristic of *P. vivax* malaria infected patients, at Mendi town, Northwest Ethiopia

Clinical features	Frequency
Age (years)	
Median age	14 (1 month to 60 years)
• <5	101 (26.3 %)
• 5–14	97 (25.26 %)
• >14	186 (48.4 %)
Sex	
• Male	202 (52.6 %)
• Female	182 (47.4 %)
Temperature	37.89 (°C)
Febrile cases	329 (85.7 %)
Blood glucose level	109.89 (mg/dL)
Hemoglobin level	12.4 (g/dL)
Anemic cases	115 (29.9 %)
Geometric mean parasite count	3745 (parasite/μL)

### Status of uncomplicated malaria symptoms in P. vivax mono-infected patients

Uncomplicated malaria symptoms observed in the current study were headache 255 (66.4 %), aching 231 (60.2 %), chills 225 (58.6 %), vomiting 179 (46.6 %), rigor 152 (39.6 %), shivering 151 (39.3 %), cramp 150 (39.1 %), nausea 147 (38.3 %), anorexia 61 (15.9 %) and diarrhea 22 (5.72 %) (Table 2). At the time of admission, about 179 (46.6 %) and 22 (5.7 %) of the patients had vomiting, and diarrhea, respectively. But, none had symptoms of splenomegaly and hepatomegaly. Although most patients, 360 (93.8 %) had a history of fever for the past 48 h, only 227 (59.1 %) were febrile or had axillary temperature ≥37.5 °C (Table 2).

**Table 2:** Frequencies of uncomplicated symptoms in *P. vivax* malaria infected patients, Mendi town, Northwest Ethiopia

Symptoms of uncomplicated malaria	Frequency (%)
Headache	255 (66.4)
Diarrhea	22 (5.7)
Chills	225 (58.6)
Aching	231 (60.2)
Vomiting	179 (46.6)
Anorexia	61 (15.9)
Nausea	147 (38.3)
Rigor	152 (39.6)
Shivering	151 (39.3)
Cramp	150 (39.1)
History of fever for about 48 h	360 (93.8)
Fever at time of enrollment (Febrile) body temperature ≥37.5 °C	227 (59.1)
Splenomegaly	0 (0)
Hepatomegaly	0 (0)

### Incidence of severe malaria symptoms

Severe malaria symptoms were observed in 55 (14.32 %) patients infected with *P. vivax* mono-infection. A total of 36 (65.5 %) patients fulfilled at least one of the WHO criteria for severe malaria symptoms while 19 (34.5 %) had two or more overlapping severity indicators. of the later cases, 12 (63.2 %) had two combined symptoms, 5 (26.3 %) had three combined symptoms and the remaining 2 (10.5 %) had four combined symptoms.

Some of these clinical manifestations were: prostration *n* = 14 (25.45 %), persistent vomiting *n* = 9 (16.36 %), respiratory distress *n* = 6 (10.9 %), hypoglycemia *n* = 5 (9.1 %), hyperpyrexia *n* = 8 (14.5 %) and severe anemia *n* = 13 (23.63 %). Major severe malaria symptoms observed were prostration, and severe anemia, in combination with other symptoms including persistent vomiting, hyperpyrexia, and hypoglycemia. However, none had signs of confusion, coma, hemoglobinuria or discoloration of urine and hypotension (Table 3). None of the participants died during the study period.

**Table 3:** Clinical manifestations of severe malaria symptoms in patients (*n* = 55) infected with *P. vivax,* Mandi town, Northwest Ethiopia

Clinical symptom	Frequency (%)
Severe anemia	13 (23.63)
Respiratory distress	6 (10.9)
Prostration	14 (25.45)
Hyperpyrexia	8 (14.5)
Persistent vomiting	9 (16.36)
Hypoglycemia	5 (9.1)
Comma	0 (0)
Convulsion	0 (0)
Hypotension	0 (0)
Hemoglobinuria	0 (0)

Most severe malaria manifestations were presented in children less than 5 years. To mention, severe anemia was observed in 12 children aged less than 5 years and 2 children aged between 5 and 14, but none among adults. These three severe symptoms were higher among children under 5 years than other age groups (Table 4).

**Table 4:** Incidence of severe malaria symptoms among different age groups of patients infected with *P. vivax* malaria, at Mandi town, Northwest Ethiopia

Clinical features	Age (year)		
	<5	5–14	>14
Respiratory distress	3 (57.9 %)	2 (31.6 %)	1 (10.5 %)
Hyperpyrexia	5 (62.5 %)	1 (12.5 %)	2 (25 %)
Persistent vomiting	5 (55.5 %)	3 (33.3 %)	1 (10 %)
Severe anemia	11 (84.6 %)	2 (15.4 %)	0 (0)
Prostration	8 (57.14 %)	4 (28.57 %)	2 (14.28 %)
Hypoglycemia	3 (60 %)	2 (40 %)	0 (0)

From the analysis made on association between parasite load and severe malaria symptoms, it was observed that differences in parasite load did not affect the occurrence of respiratory distress, hyperpyrexia, and hypoglycemia. Significant differences were not observed (*P* > 0.05) with respect to relative risk (RR) of *P. vivax* infected patients of different parasite load (between 1000 and 9999, and ≥10,000). However, severe anemia, prostration, and persistent vomiting were significantly associated (*P* < 0.05) with severe parasitemia (≥10,000 parasite/μL), [(OR = 3.8, 95 % CI, 1.1–13.7; OR = 4.4, 95 % CI, 1.4–13.9; and OR = 7. 95 % CI, 1.8–27.4), respectively (Table 5).

**Table 5:** Strength of association between parasitemia and severe malaria indicators in patients infected with *P. vivax* malaria, Mandi town, Northwest Ethiopia, 2014/15

Parasite load	Sever malaria indicators (%)											
	Severe anemia		Respiratory distress		Prostration		Persistent vomiting		Hyperpyrexia		Hypoglycemia	
	Yes	No	Yes	No	Yes	No	Yes	No	Yes	No	Yes	No
1000–9999	9 (3.4)	258 (96.6)	4 (1.5)	263 (98.5)	10 (3.7)	257 (96.3)	5 (1.9)	262 (98.1)	5 (1.9)	262 (98.1)	4 (1.5)	263 (98.5)
≥10,000	4 (11.8)	30 (88.2)	2 (5.9)	32 (94.1)	4 (12.1)	29 (85.3)	4 (11.8)	30 (88.2)	3 (8.57)	32 (94.1)	1 (2.9)	33 (97.1)
Relative risk (RR)	RR = 0.26 (95 % CI, 0.076–0.9), *P* = 0.034		RR =0.24 (95 % CI, 0.043–1.38), *P* = 0.11		RR =0.28 (95 % CI, 0.08–0.95), *P* = 0.042)		RR =0.14 (95 % CI, 0.036–0.56), *P* = 0.005		RR =0.19 (95 % CI, 0.045–0.86), *P* = 0.03		RR = 1.9 (95 % CI, 0.2–18.4), *P* = 0.54	

## Discussion

The overall prevalence of malaria during the study period (2014/15) was 29.8 %, (declined by 3.3 % from the 33.1 % prevalence documented in 2005 by the two local health facilities 2013) (Unpublished data). The overall prevalence of malaria observed during the study period was slightly lower than the recent report from southern part of Ethiopia (31.9 %) [16] and much lower than report from Nigeria (81.9 %) [23]. The possible explanation for the observed discrepancy could be due to the intense and diverse malaria control strategies undertaken in most parts of the country [22].

*P. vivax* mono-infection has been associated with severe and fatal disease in endemic areas [13, 24, 25]. The observed severe malaria symptoms in this study (14.3 %) was almost similar to reports from southern Ethiopia (13.67 %) and Tertiary care center of central India among hospitalized patients (17.2 %) due to *P. vivax* malaria [16, 18], but lower than report from Eastern Sudan’s New Halfa Hospital (27.8 %) [26]. The most commonly encountered severe malaria manifestations of *P.vivax* mono infection in this study were prostration, followed by severe anemia, persistent vomiting, hyperpyrexia, respiratory distress, and hypoglycemia. As observed in our study, severe malaria symptoms commonly detected in *P. vivax* infected patients is severe anemia [24]. This could be due to continuous presence of the parasite in liver as hypnozoite stage, infecting and destroying young RBCs [25]. Also, for every infected RBC destroyed during *P. vivax* infection, 32 non-infected RBCs are removed from the circulation, compared to the loss of 8 RBCs for every infected erythrocyte in *P. falciparum* malaria [27]. Other assumption is that anemia might occur as a result of rigor inflammatory reactions [28] and phagocytosis of non-parasitized red blood cells, increased splenic clearance, and dyserythropoiesis in bone marrow [29]. To this effect severe anemia caused by *P. vivax* is responsible for 87 % of severe diseases compared to 73 % of severe malaria complications that occur due to *P. falciparum* [13]. In this study, significant number of severe anemia patients was children aged less than 5 years. The high susceptibility of young children to severe anemia could be due to relatively faster attainment of immunity to *P. vivax* than to *P. falciparum* [30–32].

Frequency of respiratory distress due to *P. vivax* mono infection observed in this study (10.9 %) was higher than report from India (6.8 %) [18], but comparable with other report from adults living in malaria-endemic areas in Bikaner, Northwestern India (10 %) [14]. The possible mechanism of pathogenesis of respiratory distress in *P. vivax* malaria has been proposed to be severe alveolar capillary dysfunction like in *P. falciparum* [33]. This is evidenced by the comparable clinical manifestations of acute respiratory distress syndromes (ARDS) in *P. vivax* and *P. falciparum* infected patients [34–36].

Most of these symptoms are largely attributed to production of various cytokines such as TNF- α produced in response to the parasite and toxin products released during rupture of infected RBCs [37]. Also, hemozoin released from infected RBCs (iRBCs) leading to the release of pro-inflammatory cytokines that inturn induce COX-2 (cyclooxygenase-2) up-regulating prostaglandins leading to the induction of fever [38, 39]. As there is an evidence for rigor inflammatory reactions due to pro-inflammatory response and cytokines activation during *P. vivax* infection [28], the hyperpyrexia and persistent vomiting observed in this study could be due to the intense inflammatory reaction caused by *P. vivax*.

Differences in parasite load did not affect the incidence of severe malaria symptoms among assessed patients. This was in consistence with earlier report made by Price et al. [40], which explained that *P. vivax* is capable of inducing fever at levels of parasitemia lower than those causing fever in *P. falciparum* infection [40]. WHO also reported that in western Thailand, a region of low endemicity, the pyrogenic density for *P. vivax* was 180 parasites/μL compared to 1000 parasites/μL observed in *P. falciparum* infection [41]. This is mainly because of the fact that *P. vivax* has a tendency to achieve and maintain lower density parasitemia as it only invades young RBCs [42]. In addition, patients with *P. vivax* infections also tend to present all parasite stages that could be visible on the peripheral blood film [4, 43]. Hypothesis given for the lower parasitemia caused by *P. vivax* may be due to the presence of the same parasite in haemopoietic tissue than in the vascular sinus [35]. Thus, parasite load of *P. vivax* in peripheral blood could expand rigorously without its detection [44].

Naturally, *P. vivax* causes an acute febrile illness with no complications or death. However, recently reports on complications due to *P. vivax* are globally increasing [29, 45, 46]. The exact causes of changes in the clinical profile of *P. vivax* infection are uncertain. It is assumed that, it may be due to genetic alterations of the parasite or change in vector and its biting habits, indiscriminate use of anti-malarial drugs, delayed treatment, or due to declining efficacy of chloroquine or rise in chloroquine- resistant *P. vivax* strains [47–49]. In addition, prolonged existence of hypnozoite reservoir in patient’s liver could cause recurrent infection even after patients successfully treated [50]. The increasing evidence on severe malaria complications associated with *P. vivax* has implication on the current global target of malaria eradication. Using knowledge and long experience accumulated over periods on *P. falciparum,* all concerned bodies including policy makers, researchers and others working in the same field should characterize clinical epidemiology and economic burden of *P. vivax* in different geographical areas for better management of burdens due to P.vivax infection.

### Study limitation

The lack of confirmation of absence of mixed infection due to *P.vivax* and *P.falciparium* using PCR is a major limitation of this study.

## Conclusion

Some severe malaria complications such as prostration, severe anemia, respiratory distress, hyperpyrexia and persistent vomiting were observed in *P. vivax* mono-infected individuals. This strengthens the fact that parasite. *P. vivax* is no longer benign. Therefore, to meet the set international targets for reduction of malarial morbidity and mortality, and regional elimination of *P.vivax* infection, concerned bodies should no longer neglect this parasite.

## Abbreviations

ARDS: Acute respiratory distress syndromes
CI: Confidence interval
COX-2: Cyclooxygenase-2
CSA: Central Statistical Agency
Glu: Blood glucose
Hb: Hemoglobin
IRBC: Infected red blood cells
OR: Odd ratio
RR: Relative risk
